# Ferroptosis Related Immunomodulatory Effect of a Novel Extracellular Polysaccharides from Marine Fungus *Aureobasidium melanogenum*

**DOI:** 10.3390/md20050332

**Published:** 2022-05-19

**Authors:** Yuqi Lin, Jiajia Yang, Lianxiang Luo, Xiaoyong Zhang, Shengyu Deng, Xiaodan Chen, Yiyang Li, Alaa El-Din A. Bekhit, Baojun Xu, Riming Huang

**Affiliations:** 1 Guangdong Provincial Key Laboratory of Food Quality and Safety, College of Food Science, South China Agricultural University, Guangzhou 510642, China; linyuqi@stu.scau.edu.cn (Y.L.); yangjiajia@stu.scau.edu.cn (J.Y.); dengsy202013@163.com (S.D.); 18125907913@163.com (X.C.); 2 The Marine Biomedical Research Institute, Guangdong Medical University, Zhanjiang 524023, China; luolianxiang321@gdmu.edu.cn; 3 The Marine Biomedical Research Institute of Guangdong Zhanjiang, Zhanjiang 524023, China; 4 Joint Laboratory of Guangdong Province and Hong Kong Region on Marine Bioresource Conservation and Exploitation, College of Marine Sciences, South China Agricultural University, Guangzhou 510642, China; zhangxiaoyong@scau.edu.cn (X.Z.); li-yiyang0731@126.com (Y.L.); 5 Department of Food Sciences, University of Otago, P.O. Box 56, Dunedin 9054, New Zealand; aladin.bekhit@otago.ac.nz; 6 Food Science and Technology Program, Beijing Normal University−Hong Kong Baptist University−United International College, Zhuhai 519087, China; baojunxu@uic.edu.cn

**Keywords:** *Aureobasidium melanogenum*, polysaccharide, structural characterization, ferroptosis, immunomodulatory

## Abstract

Marine fungi represent an important and sustainable resource, from which the search for novel biological substances for application in the pharmacy or food industry offers great potential. In our research, novel polysaccharide (AUM-1) was obtained from marine *Aureobasidium melanogenum* SCAU-266 were obtained and the molecular weight of AUM-1 was determined to be 8000 Da with 97.30% of glucose, 1.9% of mannose, and 0.08% galactose, owing to a potential backbone of α-D-Glcp-(1→2)-α-D-Manp-(1→4)-α-D-Glcp-(1→6)-(SO_3_^−^)-4-α-D-Glcp-(1→6)-1-β-D-Glcp-1→2)-α-D-Glcp-(1→6)-β-D-Glcp-1→6)-α-D-Glcp-1→4)-α-D-Glcp-6→1)-[α-D-Glcp-4]_26_→1)-α-D-Glcp and two side chains that consisted of α-D-Glcp-1 and α-D-Glcp-(1→6)-α-D-Glcp residues. The immunomodulatory effect of AUM-1 was identified. Then, the potential molecular mechanism by which AUM-1 may be connected to ferroptosis was indicated by metabonomics, and the expression of COX2, SLC7A11, GPX4, ACSL4, FTH1, and ROS were further verified. Thus, we first speculated that AUM-1 has a potential effect on the ferroptosis-related immunomodulatory property in RAW 264.7 cells by adjusting the expression of GPX4, regulated glutathione (oxidative), directly causing lipid peroxidation owing to the higher ROS level through the glutamate metabolism and TCA cycle. Thus, the ferroptosis related immunomodulatory effect of AUM-1 was obtained.

## 1. Introduction

In recent decades, marine natural polysaccharides have received more and more attention because of their significant bioactive properties. Interestingly, fungal extracellular polysaccharides as a promising source of nutrients have attracted an increasing focus [1]. Previous reports revealed that marine fungal extracellular polysaccharides possessed structural diversity and potential biological activities, such as an *Aspergillus versicolor* extracellular polysaccharide composed of residues of (1→6)-linked α-D-glucopyranose, slightly branched by single α-D-mannopyranose residues [2], *Hansfordia sinuosae* extracellular polysaccharide mainly composed of mannose residue possessed antitumor activity [3], a polysaccharide from *Phoma herbarum* YS4108 having immunomodulatory effects on T cells and dendritic cells [4], an extracellular polysaccharide from the mangrove-associated *Fusarium oxysporum,* and the polysaccharide derived from *Arthrospira platensis* with antioxidant activity [5,6]. However, more marine fungal polysaccharides still need to be investigated to understand their structural features and mechanisms of action in depth. *Aureobasidium melanogenum* belongs to the genus *Aureobasidium*, widely distributed in many ecological resources [7]. Previous investigations mainly focused on extracellular polysaccharides of *A. melanogenum* not originating from the marine ecosystem [8,9,10], providing only a report on the polysaccharides from the marine ecological environment [11]. Research on the biological activity of *A. melanogenum* is rare, and it is worth studying if the polysaccharides from marine Scleractinia-associated fungus *A. melanogenum* will exhibit a potential biological effect, such as immunomodulatory and antioxidant activities, which will contribute to a more comprehensive understand of marine resources and a wider application of marine fungi in immunological therapy. Thus, the specific structural characterization and evaluation of their biological molecular mechanisms are urgent. In this study, the structural characterization of AUM-1 was elucidated by the UV data, FT-IR analysis, methylation analysis, and NMR spectrometry. Then, the metabolomics, metabolic targets analysis, and verification from Western blot and immunofluorescence staining were used to investigate to point out the potential mechanism of the ferroptosis related immunomodulatory effect.

## 2. Results

### 2.1. Preparation and Physicochemical Properties

**Figure 1:**
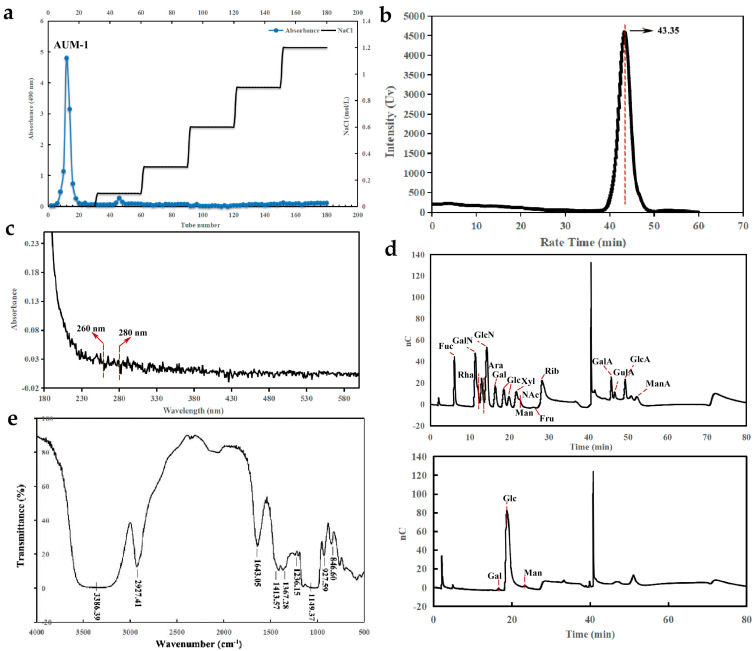
Physicochemical properties and NMR analysis of AUM-1. (**a**) HPLC chromatograms. AUM-1 was obtained through the DEAE52 ion-exchange column with the eluent of 0 M NaCl. (**b**) UV. AUM-1 contains little protein and nucleic acid as no obvious changes in the scanning wave of 260 nm and 280 nm. (**c**) The molecular weight (Mw). lgMw-RT, y = −0.1985x + 12.509, R^2^ = 0.9964. (**d**) Monosaccharide composition analysis. (**e**) FT-IR spectrum of AUM-1.

AUM-1 was obtained through the DEAE52 ion-exchange column with the eluent of the distilled water (Figure 1a). The physicochemical properties of AUM-1 were analyzed, and the total polysaccharide content and the SO_3_^−^ content of AUM-1 were determined to be 73.23% ± 2.91% and 1.74% ± 0.021% respectively. As the UV scanning shows, we concluded that the AUM-1 contains little protein and nucleic acid as no obvious changes in the scanning wave of 260 nm and 280 nm (Figure 1b). According to the HPGPC data, the molecular weight of AUM-1 is rounded to 8000 Da (Figure 1c).

### 2.2. Structural Characterization

#### 2.2.1. Monosaccharide Composition Analysis

The monosaccharide compositions analysis of AUM-1 revealed that glucose is the major monosaccharide of AUM-1 with 97.3%, and the trace monosaccharides were mannose (1.9%) and galactose (0.8%), respectively. Thus, the main backbone of AUM-1 might be composed of glucose (Figure 1d).

#### 2.2.2. FT-IR Analysis

The FT-IR spectrum showed that AUM-1 has absorption bands of polysaccharides, with the broadband at 3386 cm^−1^ assigned to the stretching vibration of O-H, and the bands at 2927 cm^−1^ represented the stretching vibration of C-H. All have been regarded as the characteristic absorption peaks of polysaccharide. The stretching vibration of C=O in AUM-1 was found at 1643 cm^−1^ [12]. The absorptions at 1413 cm^−1^ and 1149 cm^−1^ were ascribed to the stretching vibration of C-O-C [13]. The peaks at 1367 cm^−1^ and 927 cm^−1^ have respectively speculated the existence of C=O symmetrical stretching vibration and the asymmetric ring stretching vibrations of the pyranose ring [14]. The weak absorption at 1236 cm^−1^ confirmed the sulfate stretching vibrations in AUM-1 [15]. Moreover, the adjacent peak at 846 cm^−1^ was found, which has been reported to be due to sulfate groups at the axial C-4 position (Figure 1e) [16].

#### 2.2.3. Methylation Analysis

Methylation analysis results indicated that AUM-1 was composed of 2,3,4,6-Me_4_-Glcp (10.98%), 3,4,6-Me_3_-Manp (3.08%), 3,4,6-Me_3_-Glcp (2.75%), 2,3,6-Me_3_-Glcp (61.96%), 2,3,4-Me_3_-Glcp (4.82%), and 2,3-Me_2_-Glcp (16.42%) (Table 1) (Supplement Table S1). The results indicated that the 1,4-linked glucose residues were the major building blocks of AUM-1, and a small number of residues were mannose residues with the potential linkage type of 1,2-linked mannose residues. Owing to the content limitation and instrument precision, the final methylation data were analyzed without the linkage type of galactose.

#### 2.2.4. NMR Analysis

**Figure 2:**
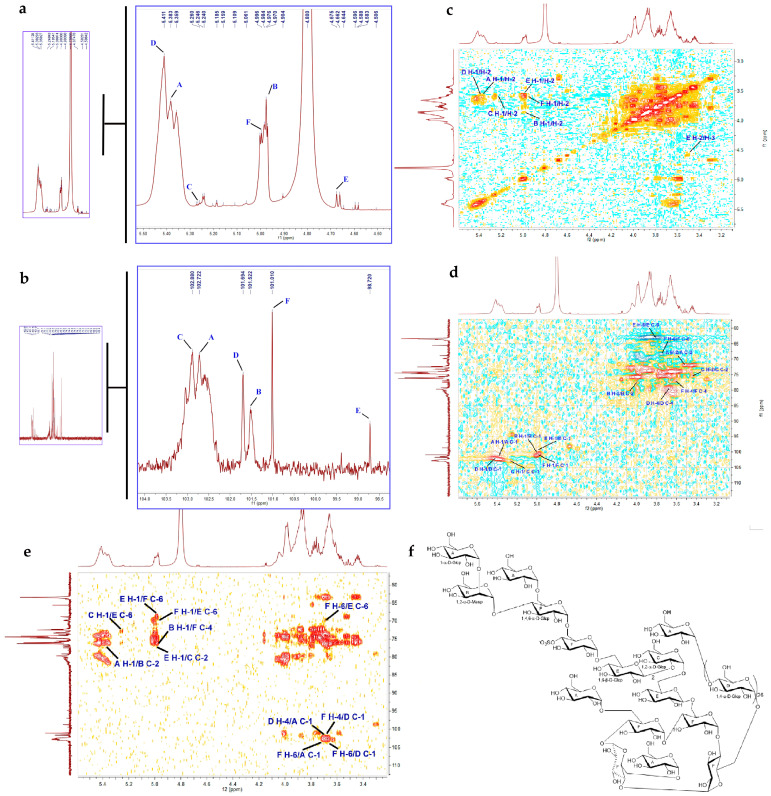
NMR data of AUM-1. (**a**) ^1^H NMR spectrometry. (**b**) ^13^C NMR spectrometry. (**c**) COSY. (**d**) HSQC. (**e**) HMBC. (**f**) Structural details of AUM-1.

To further understand the six monosaccharide residues (A–F) revealed by the methylation analysis, the sequential residue linkage of AUM-1 was carried out by NMR analysis. With the ^1^H NMR chemical shifts of six protons at δH 5.38 (H-1), 3.60 (H-2), 3.70 (H-3), 3.95 (H-4), 3.97 (H-5), and δH 3.60 (H-6) (Figure 2a), and ^13^C NMR spectrometry chemical shifts at δC 102.72 (C-1), 71.94 (C-2), 74.03 (C-3), 70.61 (C-4), 71.68 (C-5), and 62.30 (C-6) (Figure 2b), it was possible to differentiate between the separate spin systems of H-1/H-2, H-2/H-3, H-3/H-4, H-4/H-5, and H-5/H-6 in ^1^H-^1^H COSY spectrum (Figure 2c). These data supported residue A to be 1-α-D-Glcp residue [17]. The ^1^H NMR chemical shifts of six protons at δH 4.97 (H-1), 3.89 (H-2), 3.92 (H-3), 3.79 (H-4), 3.64 (H-5), and 3.75 (H-6), and chemical shifts at δC 101.52 (C-1), 76.30 (C-2), 68.95 (C-3), 65.20 (C-4), 71.77 (C-5), and 60.04 (C-6) (Figure 2d), combined with ^1^H-^1^H COSY spectrum to residue B to be 1,2-α-D-Manp residue [18]. Similarly, the ^1^H NMR and ^13^C NMR spectrometry chemical shifts together with ^1^H-^1^H COSY spectrum confirmed the residues C-F to be 1,2-α-D-Glcp [19], 1,4-α-D-Glcp [17], 1,6-β-D-Glcp [20], and 1,4,6-α-D-Glcp residues [17], respectively. Further structural assignment and sequencing of the residues A–F was accomplished based on the key HMBC correlations of H-1 (δH 5.38, A) to C-2 (δC 76.30, B), H-1 (δH 4.97, B) to C-4 (δC 76.01, F), H-1 (δH 4.99, F) to C-4 (δC 76.01, F), H-6 (δH 3.71, F) to C-6 (δC 71.40, E), H-1 (δH 4.97, E) to C-2 (δC 77.20, C), and H-4 (δH 3.68, F) to C-1 (δC 101.69, D) (Figure 2e). In addition, the sulfuric group attached to C-4 (δC 76.01, F) was discerned according to the strong deshielding properties of the sulfate substituent influencing the chemical shifts of both ^1^H and ^13^C nuclei next to the site of attachment [21], as well as the NMR data and molecular formula analysis of AUM-1. Based on the monosaccharide composition analysis, FT-IR analysis, methylation analysis, and the details of NMR data, the gross structure of AUM-1 was further concluded (Figure 2f). The four-membered carbohydrate cycle of 1,4,6-α-D-Glcp (Residue F) in the AUM-1 structure was consistent with NMR spectrometry chemical shifts, the existence sulfate groups at the axial C-4 position of 1,4,6-α-D-Glcp (Residue F) corresponded to the FT-IR results, and the Mw of this structure was close to the previous data. From the aforementioned evidence, based on all the analyzed data, ^1^H and ^13^C NMR spectrometry chemical shifts of AUM-1 were ensured (Table 2).

### 2.3. Immunomodulatory Effects of AUM-1 on RAW264.7 Cells

**Figure 3:**
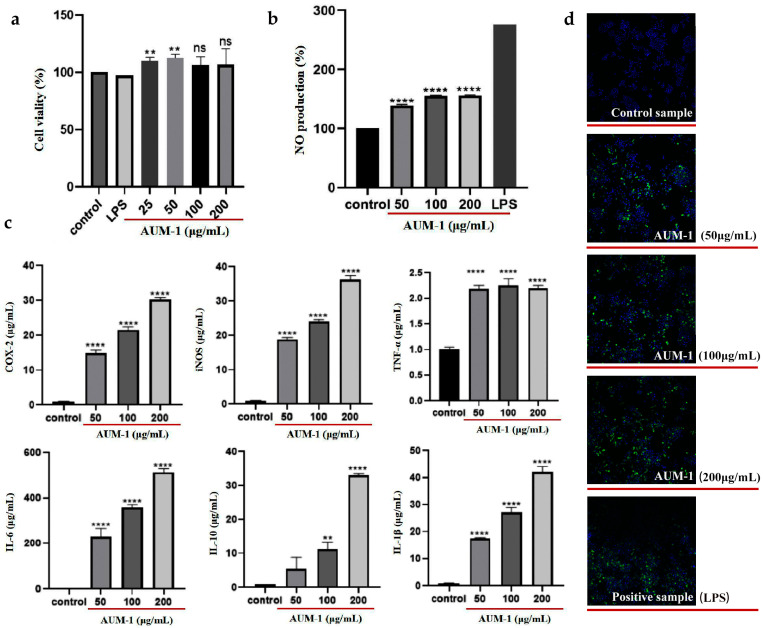
Immunomodulatory effect of AUM-1 on RAW264.7 cells. (**a**) Cytotoxicity. (**b**) NO production. (**c**) Expression of COX2, iNOS, TNF-α, IL-1β, IL-6, and IL-10. (**d**) Immunofluorescence. ** *p* < 0.05 and **** *p* < 0.001 as compared to control sample, ns means no significant.

In our study, the cytotoxicity of AUM-1 was evaluated by the cell vitality of macrophage RAW264.7 cells. As the results of the CCK-8 assay showed, the vitality rate of RAW264.7 cells showed an increasing trend above 100%, which indicated that AUM-1 in all used concentration ranges was not cytotoxic and could promote the proliferation of RAW264.7 cells (Figure 3a). Different concentrations of AUM-1 (25–200 μg/mL) all contribute to improving the production of NO, but are inferior to the positive sample (LPS) with 2.5 μg/mL (Figure 3b), which indicated that the AUM-1 exhibited inflammation-promotion activity. Generally, the expression of immune cytokines, such as COX2, iNOS, TNF-α, IL-1β, IL-6, and IL-10, is essential to assess the immune regulation of macrophages, which play an important role in immune responses and host defense [22]. The data evaluated by corresponding ELISA kits showed that the expression of mentioned immune cytokines increased in a dose-dependent manner upon treatment with AUM-1, the change of TNF-α was not obvious (Figure 3c). Moreover, the immunofluorescence detection of RAW264.7 cells was further applicated, and the AUM-1 samples with 50, 100, and 200 μg/mL showed an increasingly obvious fluorescence characteristic, which was closely changed with the LPS (Figure 3d). These results showed the immunomodulatory effect of AUM-1. 

### 2.4. Metabolites Analysis

#### 2.4.1. DAMs Data

**Figure 4:**
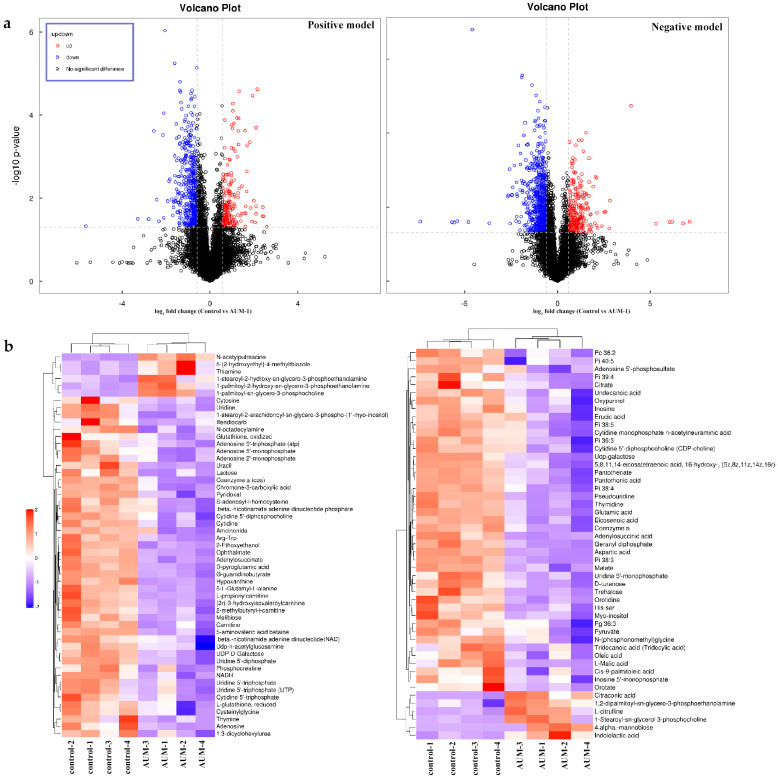
The metabolites data analysis of AUM-1 treated RAW264.7 cells. (**a**) Volcano figures of significant DAMs. (**b**) Hierarchical cluster analysis of AUM samples and control samples (4 repeating groups).

As shown in the volcano data, the differently accumulated metabolites (DAMs) were identified in both positive and negative models (Figure 4a). In the data, hierarchical cluster analysis was used to show the differences or similarities of the detected metabolites between the AUM-1 groups and the control groups. The analysis graph revealed that the differences in significant DAMs between the sample and control groups were obvious, which indicated that the detection and identification results of the significant DAMs were reasonable and reliable (Figure 4b). According to the OPLS-DA model, 102 significant DAMs with *p* < 0.05 and VIP > 1 were ensured under both positive and negative models, with 90 significantly down-regulated metabolites and 12 up-regulated metabolites (Supplement Table S2).

#### 2.4.2. KEGG Pathways Analysis

**Figure 5:**
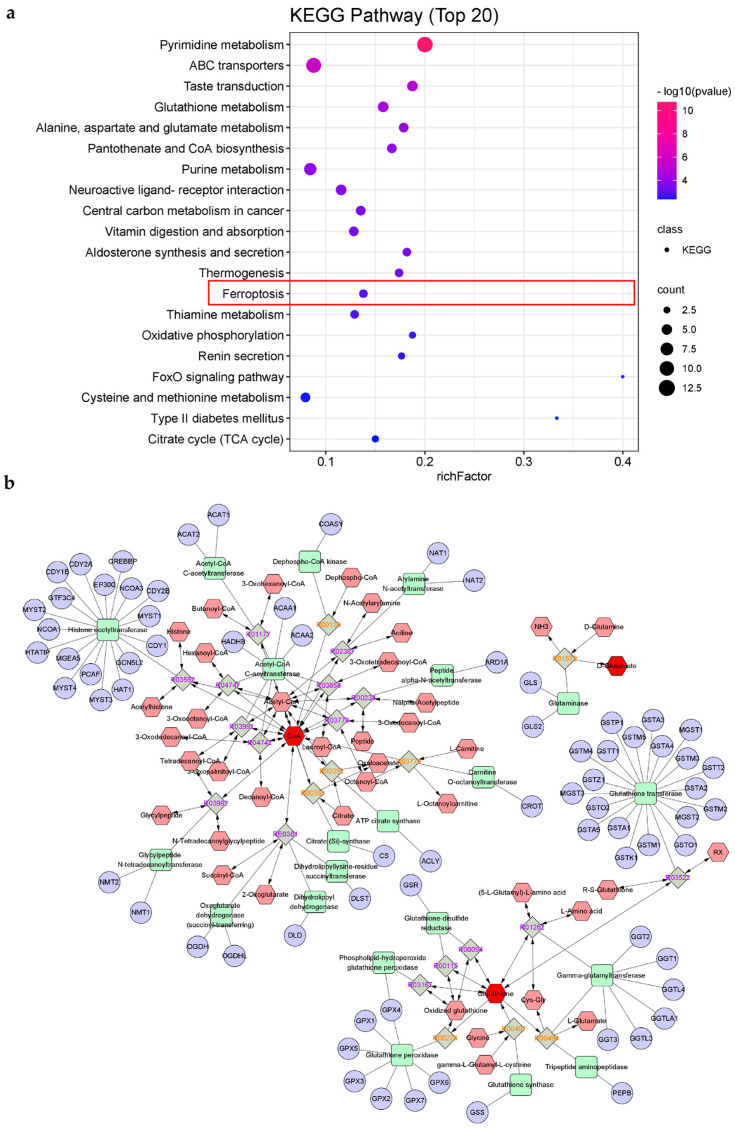
KEGG pathways of AUM-1 treated RAW264.7 cells. (**a**) Bubble diagram of the top 20 KEGG pathways, the ferroptosis pathway was emphasized in the red border. (**b**) Compound-reaction-enzyme-gene networks of the significantly DAMs in ferroptosis. The red hexagons, grey diamonds, green rectangles, and purple circles represent the metabolites, reactions, proteins, and genes, respectively.

Furthermore, 48 KEGG pathways with *p* < 0.05 were finally selected by analyzing all selected significant DAMs (Supplement Table S3), and the top 20 of them were emphasized (Figure 5a). Interestingly, the ferroptosis pathway (mmu04216) was enriched in the metabolites data, which has been pointed out to be highly connected to inflammation, and further in-depth study may accelerate the development of promising therapeutic strategies, including ferritin production inhibitors, to address inflammation [23,24]. Moreover, the pathways of glutathione metabolism and citrate cycle (TCA cycle) have been reported as indirectly connected to ferroptosis [25,26]. Consequently, we firstly speculated on the inner connection between the immunomodulatory effect and ferroptosis mechanism under the treatment of AUM-1. Moreover, the significant DAMs, glutamic acid, coenzyme A (CoA), L-glutathione (reduced), and glutathione (oxidized), were enriched in both ferroptosis pathways and glutathione metabolism, which was paid close attention. Under the analysis of Metscape software, the compound–reaction–enzyme–gene networks of the glutamic acid, coenzyme A (CoA), and glutathione (oxidized) and the associated targets were applicated (Figure 5b).

### 2.5. Verification of Ferroptosis-Related Immunomodulatory

**Figure 6:**
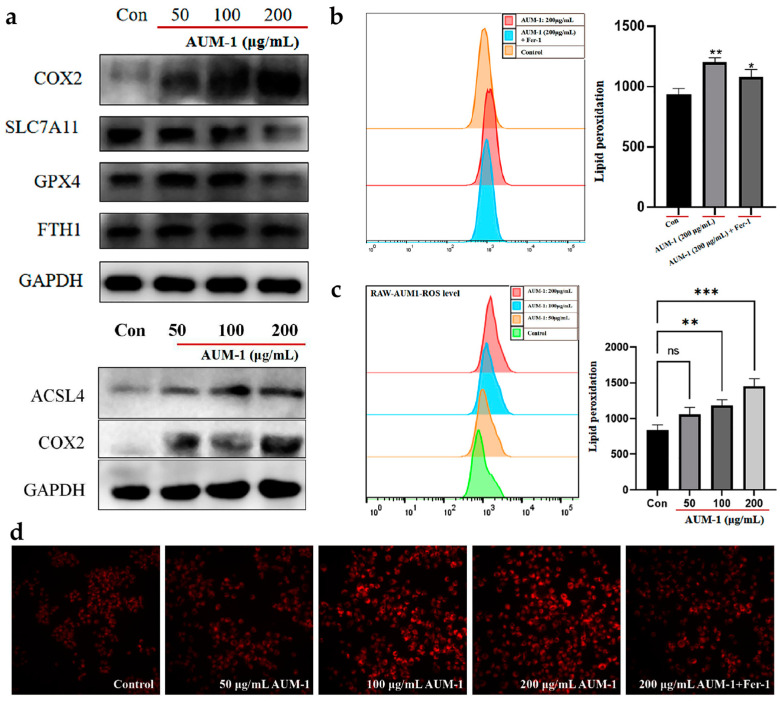
Verification of ferroptosis-related immunomodulatory effect. (**a**) Western blot results of ferroptosis related proteins. (**b**) ROS level treated with AUM-1. (**c**) ROS level treated with AUM-1 and AUM-1 + Fer-1. (**d**) Images of immunofluorescence. Fer-1, Fe^2+^ inhibitor. * *p* < 0.1, ** *p* < 0.05 and *** *p* < 0.001 as compared to control sample, ns means no significant.

To verify the occurrence of ferroptosis in the macrophage RAW264.7 cells under the treatment of AUM-1, the expression of ferroptosis characterized proteins was evaluated by Western blot analysis (Figure 6a). The up-expression of COX2 and ACSL4 and down-expression of SLC7A11 and GPX4 preliminarily revealed the process of ferroptosis [27]. In our results, the higher ROS level increased in a dose-dependent manner (Figure 6b). Su et al. have pointed out that ROS induced lipid peroxidation in apoptosis and ferroptosis [28]. Moreover, under the treatment of ferrous inhibitor-1 (Fer-1), the ROS level was inhibited while compared with the AUM-1 only, this phenomenon indicated the ROS level was affected by the Fe^2+^ from ferroptosis (Figure 6c). In addition, immunofluorescence detection was used to visually show the changes in RAW264.7 cells under the different treatments. As shown, the immunofluorescence character was more obvious with a higher concentration of AUM-1, and the added Fer-1 sample was inhibited while compared with the same concentration of the AUM-1 sample (200 μg/mL) (Figure 6d). The results confirmed that the ferroptosis proceeded in the RAW 264.7 cell under the treatment of AUM-1.

## 3. Discussion

Diversified marine fungi, and especially their extracellular polysaccharides, have received more and more attention because of their significant bioactive properties. The immune activity of polysaccharides is widely reported, but their clear structure and potential mechanisms are valuable to the development and application of marine fungi. In our study, a novel polysaccharide (AUM-1) from the marine fungi *A. melanogenum* was researched, which had a molecular weight of 8000 Da with a main monosaccharide of 97.30% of glucose, and the structure possessed an acyclic side chain consisting of α-D-Glcp-1 and α-D-Glcp-(1→6)-α-D-Glcp residues. AUM-1 showed a significant effect on the cytokines (COX2, iNOS, TNF-α, IL-1β, IL-6, and IL-10) of macrophage RAW 264.7 cells. We further combined the analysis of metabolites to clarify the immunomodulatory mechanisms of AUM-1 and the verification test of the expression of ferroptosis-related proteins (COX2, SLC7A11, GPX4, ACLS4, and FTH1) suggested the connection between immunomodulatory effects and ferroptosis. Thus, the involved metabolites in the pathway of ferroptosis, glutathione metabolism, and TCA cycle were identified from metabolites data. The glutamic acid, L-glutathione (reduced), and glutathione (oxidized) enriched in the ferroptosis pathway were further analyzed to clarify the potential mechanism. Tak et al. positioned the glutathione pathway as a central metabolic integrator in inflammatory responses related by T cells owing to the fact that glutathione-deficient T cells cannot reprogram the metabolism and the autoimmune responses were prevented [29], Alessandro et al. also supported that glutamic acid and glutamate determined immune-reproductive energy [30]. Moreover, in the results of compound–reaction–enzyme–gene networks, GPX4 was found as the metabolic target of glutathione, the down-expression from Western blot results was shown under the treatment of AUM-1, and the GPX4 downregulation is employed as a marker of ferroptosis [27,31]. Under the treatment of AUM-1, the up-regulated SLC7A11 may have contributed to inhibiting GPX4 expression through glutathione (oxidized), and this process led to an increased lipid peroxidation, thereby causing the development of ferroptosis, which was defined as the glutathione–GPX4 axis [27]. Further, the up-regulated ACLS4 that had been researched directly led to the lipid peroxidation [32], which matched the results from the Western blot. Moreover, the TCA cycle was pointed out owing to the connection between the immunomodulatory effect and ferroptosis. Glutamic acid may provide alpha-ketoglutarate (αKG) via glutaminolysis, with ATP and ROS then flowing out of the TCA cycle, and the higher lipid peroxidation leading to the ferroptosis [25]. Otherwise, the metabolic targets of glutathione (oxidized), GSS, were also pointed out to contribute to adjusting the release of glutathione and further down-regulating the expression of GPX4 to affect the ferroptosis. 

**Figure 7:**
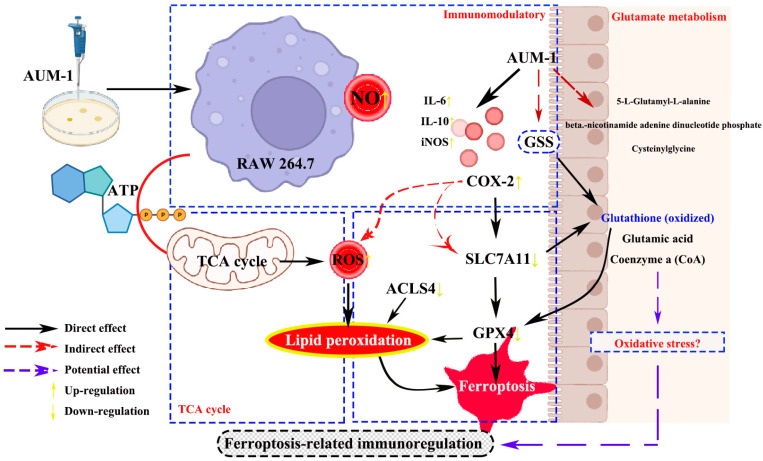
Potential mechanisms of ferroptosis-related immunomodulatory of AUM-1.

Thus, we speculated that the potential ferroptosis-related immunomodulatory mechanisms of AUM-1 may occur through the following processes: On the hand, under the treatment of AUM-1, the expression of immunity-related factors was improved to activate the immune response. On the other hand, the treatment of AUM-1 contributed to regulating GPX4 and ACLS4, directly causing the higher lipid peroxidation, and thus the occurrence of ferroptosis in RAW264.7 cells. On the other hand, the higher expression of COX2 and TCA cycle led to a higher ROS level. Thus, the occurrence of the ferroptosis-related immunomodulatory effect in the RAW264.7 cells was realized (Figure 7). Otherwise, the latest research has pointed out that the glutathione administration adjusted the oxidative stress and the inflammatory responses [33,34], which led us to further suspect the mechanism connected to the oxidization, including the inner connection of GPX4 between ferroptosis-related immunomodulatory and oxidization. However, all the conclusions need further research.

## 4. Materials and Methods

### 4.1. Materials

SCAU-266 strain was separated from the Scleractinia collected from Nansha Islands, Sansha City, Hainan Province (Hainan, China), and was identified as *A. melanogenum* with a 99% similarity (NR_159598.1) through the BLAST analysis based on the NCBI database. Potato Dextrose Agar (PDA) culture medium was purchased from Huankai Microbial Technology Co. Ltd. (Guangzhou, China). Macroporous resin NAK-9, DEAE52 cellulose, LPS and Spiro[isobenzofuran-1(3H),9′-[9H]xanthen]-3-one,3′,6′-bis(acetyloxy)-2′,7′-dichloro-(DCFH-DA) were purchased from Yuanye Bio-Technology Co. LTD (Shanghai, China). Glucose (Glc), fucose (Fuc), mannose (Man), ribose (Rib), galacturonic acid (GalA), galactose (Gal), glucuronic acid (GlcA), arabinose (Ara), xylose (Xyl), glucosamine hydrochloride (GlcN) and fructose (Fru) were provided by Boruitang Technology Co., Ltd. (Hangzhou, China). Macrophage RAW 264.7 cells stored in liquid nitrogen were purchased from Jinan University (Guangzhou, China). Dulbecco Modified Eagle Medium (DMEM), cell Lysis buffer, TBST, and antibodies was purchased from Thermo Fisher Scientific Inc (Thermo Fisher, Waltham, MA, USA). Phosphate buffer solution (PBS), penicillin-streptomycin, and fetal bovine serum (FBS) were supplied by Gibco Life Technologies (Grand Island, NY, USA). Cell Counting Kit-8 (CCK-8) was purchased from Dongren Chemical Technology Co., Ltd. (Dongying, China). Nitric oxide (NO) kit was purchased from Beyotime Biotechnology Co., Ltd. (Shanghai, China). Mouse COX2, iNOS, TNF-α, IL-10, IL-6, and IL-1β ELISA kits were purchased from NeoBioscience Biotechnology Co., Ltd. (Beijing, China), and Fe^2+^ inhibitor was purchased from Huankai Microbial Technology Co., Ltd. (Guangzhou, China). Other mentioned chemical materials were all purchased from Guangzhou Chemical Co., Ltd. (Guangzhou, China). All other reagents used in our research were of analytical grade.

### 4.2. Preparation of Polysaccharides

The SCAU-266 strain was cultivated in the PDA medium and further cultivated in an electrothermal incubator at 28 °C. When fully grown, the strain was inoculated into the liquid medium, which included the maltose 20.0 g/L, glucose 10.0 g/L, mannitol 20.0 g/L, sodium glutamate 10.0 g/L, magnesium sulfate heptahydrate 0.3 g/L, monopotassium phosphate 0.5 g/L, corn steep liquor 1.0 g/L, yeast extract 3.0 g/L, and sea salt 30.0 g/L. Then, shake cultivation was conducted at 28 °C, 130 rpm. After collecting the cultivated liquid, the existent mycelium was filtrated and expressed to one third of the original volume. Alcohol precipitation was adopted to collect the sediment, which was then deproteinated by the Sevag method. Then, after layering by centrifugation (12,000 r/min, 10 min), dissolving in deionized water (cut-off Mw 3000 Da), and freeze-drying, the crude polysaccharide was obtained. Next, crude polysaccharides were further separated and purified through macroporous resin (NAK-9) and DEAE-52 cellulose (2.6 cm × 50 cm) with an eluent of distilled water, 0.1, 0.3, 0.6, 0.9, 1.2, and 2.0 M NaCl in turn. Finally, the purified polysaccharides were obtained and signed as AUM-1, and the polysaccharide content and sulfate esters content of AUM-1 were respectively measured by the phenol-sulfuric acid method [35] and the barium sulfate turbidimetry method [36].

### 4.3. Physicochemical Characterizations

#### 4.3.1. Molecular Weight and Monosaccharide Composition

The molecular weight of AUM-1 (5 mg/mL) was determined using an LC-10A high-performance liquid chromatography (Shimadzu, Japan) with a BRT105-104-102 tandem gel column (8.0 mm × 30.0 cm). Hence, 0.05 M NaCl was used as the eluent (0.6 mL/min) according to the reported method. Monosaccharide composition analysis of AUM-1 (10 mg) was performed on an ion chromatograph ICS5000 (ThermoFisher, Waltham, MA, USA). AUM-1 was hydrolyzed by trifluoroacetic acid (TFA, 3 M) (120 °C, 3 h) based on the previously reported procedure [37].

#### 4.3.2. UV and FT-IR Spectrum

AUM-1 (30 mg/mL) was analyzed using an Evolution UV-VIS spectrometer (ThermoFisher, Waltham, MA, USA) (200–500 nm) to identify the presence of nucleic acids and proteins. AUM-1 (2 mg) compacted with grade KBr powder (200 mg) was analyzed by an FT-IR 650 spectrometer (Gangdong, China).

#### 4.3.3. Methylation

Methylation analysis of AUM-1 (2–3 mg) was carried out with a GC-MS QP2010 Ultra system (SHIMADZU, Kyoto, Japan) equipped with an RXI-5 SIL MS capillary column (30 m × 0.25 mm × 0.25 μm), according to the reported methods [38].

#### 4.3.4. NMR Analysis

AUM-1 (60 mg/mL) was dissolved in D_2_O and further freeze-dried two times to exchange deuterium. Then, 1D NMR (^1^H NMR, ^13^C NMR) and 2D NMR (^1^H-^1^H COSY, HSQC, HMBC) spectra were detected by a Bruker AVANCE IIIHD 600M spectrometer (Bruker, Zurich, Switzerland).

### 4.4. Immunomodulatory Effect

#### 4.4.1. Cell Culture and CCK-8 Assay

DMEM supplemented with 10% (*v/v*) FBS and 1% (*v/v*) penicillin-streptomycin added to macrophage RAW264.7 cells were cultured in a humidified atmosphere (5% CO_2_, 37 °C). CCK-8 assay was used to measure the viability of RAW264.7 cells. Further experiments were carried out when the sterile tissue culture flasks were filled with cells [37].

#### 4.4.2. NO Assay and Cytokine Production

The NO assay and cytokine production of RAW264.7 cells was measured according to the reported methods [37]. In short, RAW264.7 cells at logarithmic growth stage were counted. The cell suspension concentration was adjusted to 3 × 10^5^ cells/mL, 500 μL per well, and incubated at 37 °C for 24 h. The supernatant was discarded and washed with PBS. Then, 1 mL of DMEM solution (control sample), AUM-1 with 0, 50, 100, and 200 μg/mL, and LPS with 2.5 μg/mL (positive sample) were added. After incubation at 37 °C for 24 h, 50 μL of samples were added to each well, before 50 μL of Griess reagent І and 50 μL of Griess reagent II were added, absolutely shaken, and mixed. Then, enzyme marker (VersaMax, Melecular Devices, Silicon Valley, CA, USA) was used to detect the absorbance value at 540 nm. The RAW264.7 cells were treated with AUM-1 of 0, 50, 100, and 200 μg/mL, where 2.5 μg/mL of LPS was the positive control group. The levels of COX2, iNOS, TNF-α, IL-6, IL-10, and IL-1β in the supernatants of the RAW264.7 cells were measured by ELISA kits.

#### 4.4.3. Immunofluorescence Staining

RAW264.7 cells fixed with 4% paraformaldehyde for 20 min at 4 °C were incubated in 0.5% Triton for 15 min. After blocking the cells with 5% goat serum at room temperature for 30 min, the cells were later cultured with anti-hepcidin (Abcam, Cambridge, UK) at 4 °C for 24 h. The cells were incubated with a secondary antibody (Proteintech, Chicago, IL, USA) for 1 h. After washing 3 times with PBS, DAPI was applied to stain the nucleus. Immunofluorescent signals were detected under a fluorescence microscope (Thermo Fisher Scientific, Waltham, MA, USA) [39]. 

### 4.5. Metabolites Analysis

The metabolites analysis of the control and AUM-1 treated groups of the macrophage RAW264.7 cells was performed with a standard metabolic operating procedure [40,41]. ACQUIY UPLC BEH Amide column (2.1 mm × 100 mm, 1.7 µm) (Waters, MA, USA) was used to separate the samples. Further information on the obtained metabolites was analyzed through pareto-scaled principal component analysis (PCA) and orthogonal partial least-squares discriminant analysis (OPLS-DA). The analysis of variable importance in projection (VIP) value was performed based on the result of OPLS-DA. Normally, the metabolites with a standard of 0.05 < *p* < 0.1 were considered as differently accumulated metabolites (DAMs), and metabolites with the *p* < 0.05 and VIP > 1 were considered as significant DAMs. KEGG pathways of all identified DAMs were selected according to *p* < 0.05. The plug-in of Cytoscape (Version 3.9.1, https://cytoscape.org/ access on 10 April 2022), Metscape, was applicated to analyze the compound–reaction–enzyme–gene networks of the key metabolites and targets.

### 4.6. Western Blot Analysis 

The AUM-1 treated RAW264.7 cells were collected and the Western blot was conducted according to the method described previously [42]. In short, 2 × 10^6^ cells were cultured in 6-well plates for Western blots. Cell Lysis buffer was used to lyse RAW 264.7 cells and the supernatant was collected after the centrifugation (4 °C, 12,000 rpm, 10 min). Then, the RIPA lysis buffer was added to the supernatant and boiled to accomplish the protein deformation (100 °C, 10 min), before storing at −20 °C. The sample and marker (3 μL) were electrophoresed on a running gel (30% Acr-Bis, 10% APS, and TEMED). The protein was transferred to nitrocellulose membranes. Then, the transcribed membranes with protein blots were incubated in 1 × TBST containing 4% milk for 1 h at room temperature. The primary antibody was added after cutting the strips according to the molecular weight size and incubated overnight at 4 °C. After removal, the membrane was washed with TBST and incubated with anti-rabbit antibodies at room temperature for 1 h. Finally, the membrane was washed with TBST (3 times, 10 min) and exposed under the chemiluminescence (Amersham Imager 600, GE Healthcare). All primary protein expressions in our study were COX2, SLC7A11, ACLS4, GPX4, FTH1, and GAPDH. 

### 4.7. Determination of ROS and Immunofluorescence

Reactive oxygen species (ROS) was measured according a previous report described with modifications [43]. Fluorescence intensity was measured by using a fluorescence microplate reader (FilterMax F5, Molecular Devices, Silicon Valley, CA, USA). The immunofluorescence detection of adding Fe^2+^ in RAW264.7 cells corresponded with the previous methods [39].

### 4.8. Statistical Analysis

Data were shown as mean average ± standard deviations (SD) (*n* = 3). Data in all the bioassays were evaluated by Student’s *t*-test or ANOVA followed by post hoc analysis. A *p*-value < 0.05 or *p*-value < 0.01 were respectively regarded as significant differences or extremely significant differences. ** *p* < 0.05 and **** *p* < 0.001 as compared to control sample, ns means no significant.

## 5. Conclusions

In summary, a novel polysaccharide (AUM-1) from the marine fungi *A. melanogenum* had a molecular weight rounded to 8000 Da with a main monosaccharide of 97.30% of glucose, whose structure possessed an acyclic side chain consisting of α-D-Glcp-1 and α-D-Glcp-(1→6)-α-D-Glcp residues. In our previous study, AUM-1 showed a significant effect on the cytokines (COX2, iNOS, TNF-α, IL-1β, IL-6, and IL-10) of macrophage RAW 264.7 cells. The top 20 KEGG pathways from metabonomics were analyzed and the ferroptosis was consistent with those revealed by the metabonomics. Furthermore, ferroptosis characteristic factors (COX2, SLC7A11, GPX4, FTH1, ACLS4, and ROS level), including the connected metabolic targets of significant DAMs that existed in ferroptosis, were verified. Thus, we speculated that AUM-1 has a potential effect on the ferroptosis-related immunomodulatory effect in RAW 264.7 cells. Our present findings provided basic scientific research for the further investigation of new potential molecular mechanisms of marine fungal polysaccharides.

## Figures and Tables

**Table 1:** Methylation Analysis of AUM-1.

Retention Time (min)	Methylated Sugars	Molar Ratios (%)	Mass Fragments (m/z)	Linkage Type
16.330	2,3,4,6-Me4-Glcp	10.98	43, 101, 117, 129, 145, 161, 205	Glcp-1
20.630	3,4,6-Me3-Manp	3.08	43, 87, 99, 101, 129, 161, 189	Manp-1-2
20.864	3,4,6-Me3-Glcp	2.75	43, 87, 99, 117, 129, 161, 189	Glcp-1-2
21.270	2,3,6-Me3-Glcp	61.96	43, 87, 99, 117, 129, 161,173, 233	Glcp-1-4
22.724	2,3,4-Me3-Glcp	4.82	43, 99, 117, 129, 161, 189, 233	Glcp-1-6
27.453	2,3-Me2-Glcp	16.42	43, 99, 117, 159, 201,261	Glcp-1-4-6

**Table 2:** ^1^H and ^13^C NMR spectrometry chemical shifts of AUM-1.

Chemical Shift, δ (ppm)							
Glycosyl Linkage	Residue	H1	H2	H3	H4	H5	H6
		C1	C2	C3	C4	C5	C6
1-α-D-Glcp	A	5.38	3.60	3.70	3.95	3.97	3.60
		102.72	71.94	74.03	70.61	71.68	62.30
1,2-α-D-Manp	B	4.97	3.89	3.92	3.79	3.64	3.75
		101.52	76.30	68.95	65.20	71.77	60.04
1,2-α-D-Glcp	C	5.26	3.60	3.72	3.29	3.62	3.92
		102.88	77.20	71.33	73.94	63.84	62.32
1,4-α-D-Glcp	D	5.41	3.62	3.73	3.68	3.93	3.69
		101.69	71.96	72.22	79.68	71.08	60.86
1,6-β-D-Glcp	E	4.97	3.56	4.61	3.51	3.94	3.64
		98.72	70.70	73.40	70.40	71.33	71.40
1,4,6-α-D-Glcp	F	4.99	3.57	3.65	3.68	3.85	3.71
		101.01	75.73	74.02	76.01	74.60	68.47

## Data Availability

Not applicable.

## Supplementary Materials

The following supporting information can be downloaded at: https://www.mdpi.com/article/10.3390/md20050332/s1. Supplement Table S1: EI-MS spectra of the methylated sugars; Supplement Table S2: Details of 102 significant DAMs, Supplement Table S3: 48 KEGG pathways from metabolites data.
