# Supplementary material for: Ferroptosis Related Immunomodulatory Effect of a Novel Extracellular Polysaccharides from Marine Fungus Aureobasidium melanogenum
Source: Mar Drugs. 2022 May 19;20(5):332. doi: 10.3390/md20050332 (PMC9144548; doi:10.3390/md20050332)

Supplementary Table S1: EI-MS spectra of the methylated sugars

Methylation analysis of AUM-1

Retention time (min)	Methylated sugars	Molar ratios (%)	Mass fragments (m/z)	Linkage type
16.330	2,3,4,6-Me4-Glcp	10.98	43, 101, 117, 129, 145, 161, 205	Glcp-1
20.630	3,4,6-Me3-Manp	3.08	43, 87, 99, 101, 129, 161, 189	Manp-1-2
20.864	3,4,6-Me3-Glcp	2.75	43, 87, 99, 117, 129, 161, 189	Glcp-1-2
21.270	2,3,6-Me3-Glcp	61.96	43, 87, 99, 117, 129, 161, 173, 233	Glcp-1-4
22.724	2,3,4-Me3-Glcp	4.82	43, 99, 117, 129, 161, 189, 233	Glcp-1-6
27.453	2,3-Me2-Glcp	16.42	43, 99, 117, 159, 201, 261	Glcp-1-4-6

EI-MS spectra of the methylated sugars

A: 2,3,4,6-Me4-Glcp

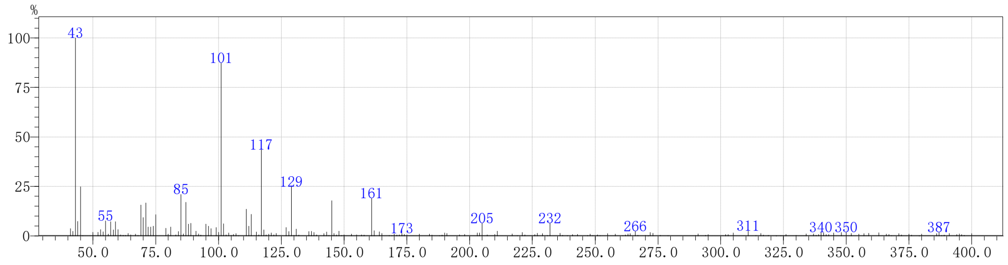

B: 3,4,6-Me3-Manp

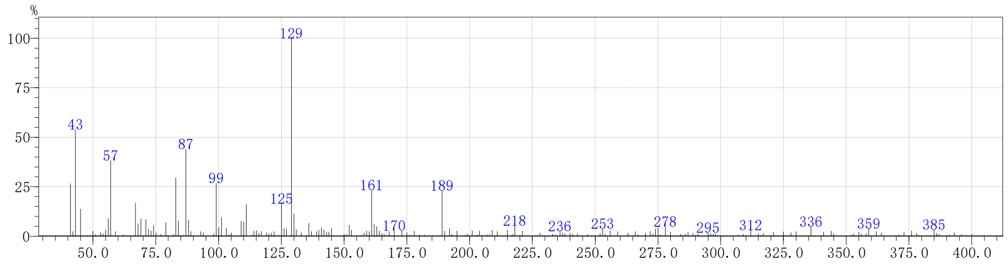

C: 3,4,6-Me3-Glcp

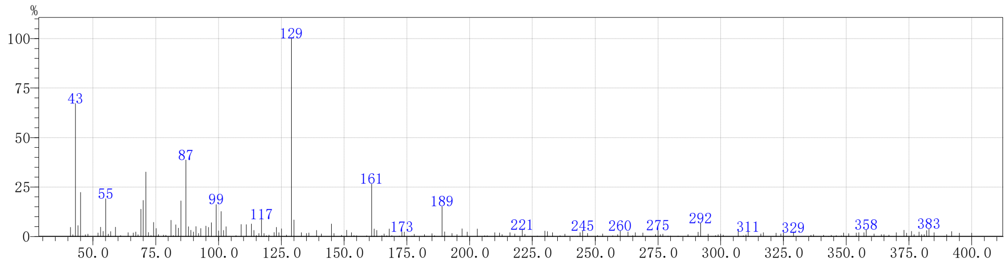

D: 2,3,6-Me3-Glcp

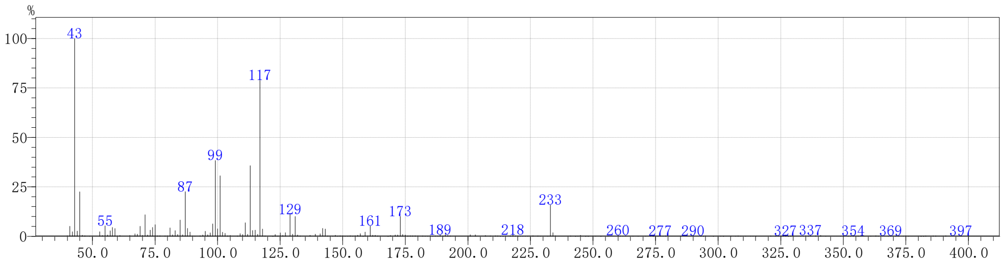

E: 2,3,4-Me_3-Glcp

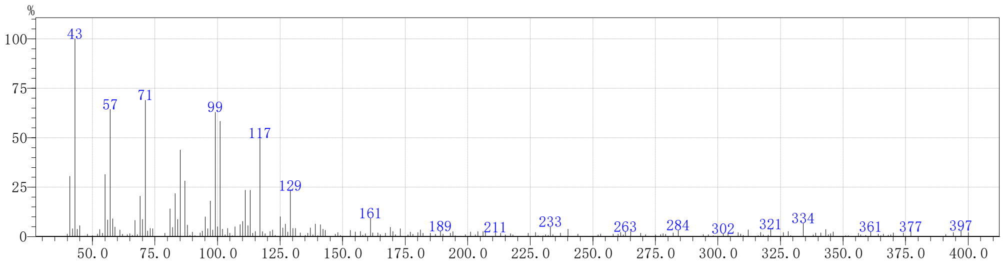

F: 2,3-Me_2-Glcp

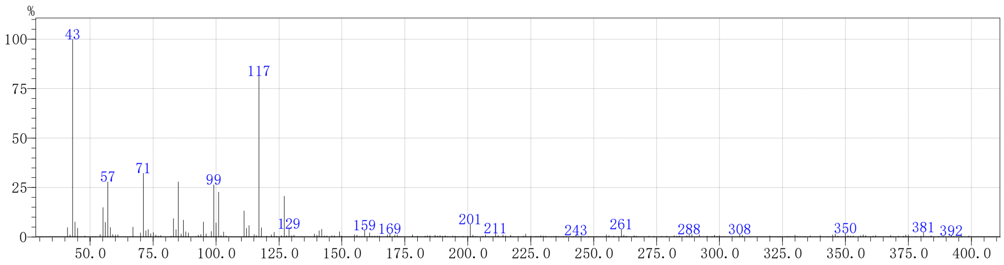

Supplement: Supplementary file 1 [file marinedrugs-20-00332-s001.zip › Supplement Table S1.pdf]
