# Supplementary material for: Ferroptosis Related Immunomodulatory Effect of a Novel Extracellular Polysaccharides from Marine Fungus Aureobasidium melanogenum
Source: Mar Drugs. 2022 May 19;20(5):332. doi: 10.3390/md20050332 (PMC9144548; doi:10.3390/md20050332)
Supplement: Supplementary file 1 [file marinedrugs-20-00332-s001.zip › Supplement Table S2 .pdf]

Supplementary Table S2

The details of 102 significant DAMs (Positive and negative models)

Name	VIP	Fold change	p-value
Aspartic acid	3.8823	0.2648	<0.001
Pi 38:3	4.7552	0.5183	<0.001
Pseudouridine	8.4153	0.4298	<0.001
Glutamic acid	3.2771	0.6450	0.0002
Adenylosuccinic acid	1.1468	0.4843	0.0002
Geranyl diphosphate	2.7274	0.2343	0.0002
Malate	3.4694	0.5156	0.0003
Thymidine	2.3622	0.5302	0.0007
Eicosenoic acid	3.5517	0.6212	0.0009
Pantothenate	4.6157	0.7221	0.0013
Trehalose	1.3501	0.6040	0.0014
Pi 38:4	5.4099	0.5709	0.0014
Udp-galactose	7.5115	0.5585	0.0015
Coenzyme a	1.3075	0.5850	0.0015
Pantothenic acid	3.0371	0.5973	0.0017
5,8,11,14-eicosatetraenoic acid, 16-hydroxy-, (5z,8z,11z,14z,16r)-	3.4126	0.6508	0.0018
Citraconic acid	5.8519	1.6817	0.0018
D-turanose	1.1070	0.5924	0.0023
Orotidine	1.6813	0.5409	0.0029
His-ser	2.9599	0.4502	0.0029
L-Malic acid	2.7054	0.3377	0.0048
Myo-inositol	2.8573	0.5869	0.0065
Pi 40:5	1.9402	0.7107	0.0066
Uridine 5'-monophosphate	5.7428	0.5691	0.0072
Pc 36:2	2.2439	0.8491	0.0073
Undecanoic acid	1.3103	0.7411	0.0075
L-citrulline	1.5105	1.6158	0.0088
Pi 38:5	3.4335	0.6193	0.0095
Indolelactic acid	2.8777	1.3306	0.0099
Pi 39:4	1.0285	0.6654	0.0103
1,2-dipalmitoyl-sn-glycero-3-phosphoethanolamine	2.4122	1.9225	0.0125
Oxypurinol	3.2597	0.6543	0.0127
Inosine	2.9035	0.6666	0.0145
Cytidine monophosphate n-acetylneuraminic acid	2.5036	0.7345	0.0155

N-(phosphonomethyl)glycine	2.7476	0.7936	0.0181
Oleic acid	10.1207	0.7167	0.0220
Pi 36:3	2.4928	0.7154	0.0222
Cytidine 5'-diphosphocholine (CDP-choline)	1.3462	0.7719	0.0230
1-Stearoyl-sn-glycerol 3-phosphocholine	1.1206	1.3432	0.0277
4.alpha.-mannobiose	1.8954	39.1696	0.0284
Citrate	2.3145	0.4492	0.0349
Pg 36:3	6.5624	0.7360	0.0366
Pyruvate	1.7049	0.7261	0.0400
Inosine 5'-monophosphate	1.7102	0.7718	0.0420
Adenosine 5'-phosphosulfate	3.9118	0.5741	0.0423
Orotate	1.1007	0.2993	0.0426
Tridecanoic acid (Tridecylic acid)	1.0364	0.8608	0.0430
Cis-9-palmitoleic acid	3.1584	0.7676	0.0474
Erucic acid	1.6224	0.8124	0.0476
G-guanidinobutyrate	5.4118	0.5681	0.0000
Adenylosuccinate	1.1996	0.3993	0.0000
Hypoxanthine	15.2589	0.5072	0.0001
2-Ethoxyethanol	1.5682	0.6646	0.0001
D-pyroglutamic acid	2.1780	0.5781	0.0001
N-acetylputrescine	1.1322	2.4538	0.0002
5-aminovaleric acid betaine	1.6404	0.6153	0.0003
(2r)-3-hydroxyisovaleroylcarnitine	1.0762	0.5040	0.0004
Uridine 5'-diphosphate	2.9849	0.6643	0.0004
5-L-Glutamyl-L-alanine	1.7223	0.4231	0.0004
Ophthalmate	1.2846	0.4692	0.0005
Pyridoxal	1.3448	0.5449	0.0005
Coenzyme a (coa)	1.5454	0.5392	0.0006
Arg-Trp	1.2697	0.4400	0.0006
UDP-D-Galactose	2.9627	0.6718	0.0007
Chromone-3-carboxylic acid	1.0005	0.5724	0.0008
L-propionylcarnitine	5.1156	0.3717	0.0011
Cytidine	6.8474	0.4408	0.0012
Uracil	4.2691	0.3988	0.0012

Melibiose	1.3090	0.5783	0.0021
Carnitine	10.5092	0.5142	0.0028
NADH	1.3381	0.4547	0.0030
N-octadecylamine	2.0898	0.2753	0.0039
Uridine	1.5552	0.4632	0.0043
Lactose	1.4493	0.6600	0.0051
2-methylbutyryl-l-carnitine	3.2970	0.5934	0.0059
Cytidine 5'-diphosphocholine	3.6007	0.7160	0.0071
Adenosine	3.2089	0.3644	0.0079
1-stearoyl-2-arachidonoyl-sn-glycero-3-phospho-(1'-myo-inositol)	1.2205	0.6641	0.0091
S-adenosyl-l-homocysteine	1.4229	0.5302	0.0096
1-palmitoyl-2-hydroxy-sn-glycero-3-phosphoethanolamine	1.4861	1.8014	0.0126
.beta.-nicotinamide adenine dinucleotide phosphate	2.6863	0.6745	0.0137
Adenosine 5'-monophosphate	5.8866	0.5662	0.0152
Cysteinylglycine	2.8585	0.6569	0.0156
Amcinonide	1.2350	0.3170	0.0166
1,3-dicyclohexylurea	2.4729	0.8261	0.0177
Udp-n-acetylglucosamine	6.0559	0.7916	0.0196
L-glutathione, reduced	11.9487	0.6449	0.0210
Thymine	1.6029	0.5165	0.0213
Adenosine 2'-monophosphate	1.1577	0.3950	0.0236
.beta.-nicotinamide adenine dinucleotide(NAD)	3.6070	0.7796	0.0250
1-palmitoyl-sn-glycero-3-phosphocholine	5.3335	1.4942	0.0299
Cytidine 5'-triphosphate	2.3235	0.6112	0.0310
Phosphocreatine	1.8885	0.5111	0.0341
Uridine 5'-triphosphate	2.5685	0.6434	0.0358
1-stearoyl-2-hydroxy-sn-glycero-3-phosphoethanolamine	1.8646	1.5997	0.0363
Adenosine 5'-triphosphate (atp)	5.1222	0.6063	0.0386

Uridine 5'-triphosphate (UTP)	1.7557	0.6505	0.0400
5-(2-hydroxyethyl)-4-methylthiazole	1.5758	1.8515	0.0410
Bendiocarb	1.0809	0.7118	0.0427
Thiamine	2.7538	1.9121	0.0446
Glutathione, oxidized	3.6410	0.7163	0.0448
Cytosine	5.5860	0.5249	0.0464

Note: 12 up-regulated metabolites were colored in red.
