# Supplementary material for: Ferroptosis Related Immunomodulatory Effect of a Novel Extracellular Polysaccharides from Marine Fungus Aureobasidium melanogenum
Source: Mar Drugs. 2022 May 19;20(5):332. doi: 10.3390/md20050332 (PMC9144548; doi:10.3390/md20050332)
Supplement: Supplementary file 1 [file marinedrugs-20-00332-s001.zip › Supplement Table S3.pdf]

Supplementary Table S3

48 KEGG pathways from metabolites data

Number	Map_ID	Map_Name	p-value	FDR
1	mmu00240	Pyrimidine metabolism	1.86489E-11	2.20057E-09
2	mmu02010	ABC transporters	1.77494E-06	0.000104721
3	mmu04742	Taste transduction	1.07816E-05	0.000424074
4	mmu00480	Glutathione metabolism	3.03708E-05	0.000895939
5	mmu00250	Alanine, aspartate and glutamate metabolism	7.92752E-05	0.001870895
6	mmu00770	Pantothenate and CoA biosynthesis	0.000112029	0.002203245
7	mmu00230	Purine metabolism	0.000146306	0.002466295
8	mmu04080	Neuroactive ligand-receptor interaction	0.000186587	0.002752161
9	mmu05230	Central carbon metabolism in cancer	0.000313192	0.004106301
10	mmu04977	Vitamin digestion and absorption	0.000403194	0.004343012
11	mmu04925	Aldosterone synthesis and secretion	0.000404857	0.004343012
12	mmu04714	Thermogenesis	0.000483996	0.004759295
13	mmu04216	Ferroptosis	0.001204406	0.010932303
14	mmu00730	Thiamine metabolism	0.001556256	0.013117019
15	mmu00190	Oxidative phosphorylation	0.002095445	0.016484164
16	mmu04924	Renin secretion	0.002514418	0.018224423
17	mmu04068	FoxO signaling pathway	0.002625552	0.018224423
18	mmu00270	Cysteine and methionine metabolism	0.003620044	0.023731396
19	mmu04930	Type II diabetes mellitus	0.003896172	0.024000858
20	mmu00020	Citrate cycle (TCA cycle)	0.004067942	0.024000858
21	mmu05012	Parkinson disease	0.004690029	0.025155611
22	mmu04918	Thyroid hormone synthesis	0.004690029	0.025155611
23	mmu04152	AMPK signaling pathway	0.005366635	0.027533173
24	mmu00220	Arginine biosynthesis	0.006099031	0.029986904
25	mmu00052	Galactose metabolism	0.006697661	0.031109444
26	mmu05032	Morphine addiction	0.007118263	0.031109444
27	mmu04211	Longevity regulating pathway	0.007118263	0.031109444
28	mmu04922	Glucagon signaling pathway	0.008642103	0.03642029
29	mmu00330	Arginine and proline	0.008981231	0.036544318

		metabolism		
30	mmu01100	Metabolic pathways	0.010797494	0.041290701
31	mmu05034	Alcoholism	0.011197478	0.041290701
32	mmu04022	cGMP-PKG signaling pathway	0.011197478	0.041290701
33	mmu00760	Nicotinate and nicotinamide metabolism	0.012522819	0.044778566
34	mmu04020	Calcium signaling pathway	0.013540191	0.046992427
35	mmu00410	beta-Alanine metabolism	0.01536141	0.051268004
36	mmu04721	Synaptic vesicle cycle	0.016075561	0.051268004
37	mmu04911	Insulin secretion	0.016075561	0.051268004
38	mmu01230	Biosynthesis of amino acids	0.018555058	0.055450392
39	mmu00630	Glyoxylate and dicarboxylate metabolism	0.018789082	0.055450392
40	mmu00471	D-Glutamine and D-glutamate metabolism	0.018796743	0.055450392
41	mmu04923	Regulation of lipolysis in adipocytes	0.021697063	0.062445207
42	mmu04066	HIF-1 signaling pathway	0.02477001	0.069591933
43	mmu04964	Proximal tubule bicarbonate reclamation	0.031408542	0.084232
44	mmu01523	Antifolate resistance	0.031408542	0.084232
45	mmu04931	Insulin resistance	0.038663417	0.101384072
46	mmu01200	Carbon metabolism	0.039610007	0.10160828
47	mmu05211	Renal cell carcinoma	0.048950411	0.120336428
48	mmu05410	Hypertrophic cardiomyopathy (HCM)	0.048950411	0.120336428

Note: Top 20 KEGG pathways were colored in red.
